# Power Graphs of Finite Groups Determined by Hosoya Properties

**DOI:** 10.3390/e24020213

**Published:** 2022-01-29

**Authors:** Fawad Ali, Bilal Ahmad Rather, Anwarud Din, Tareq Saeed, Asad Ullah

**Affiliations:** 1 Institute of Numerical Sciences, Kohat University of Science & Technology, Kohat 26000, Pakistan; fawadali@math.qau.edu.pk; 2 Mathematical Sciences Department, College of Science, United Arab Emirates University, Al Ain P.O. Box 15551, United Arab Emirates; bilalahmadrr@gmail.com; 3 Department of Mathematics, Sun Yat-sen University, Guangzhou 510275, China; anwarud@mail.sysu.edu.cn; 4 Nonlinear Analysis and Applied Mathematics (NAAM)-Research Group, Department of Mathematics, Faculty of Science, King Abdulaziz University, Jeddah 21589, Saudi Arabia; 5 Department of Mathematical Sciences, University of Lakki Marwat, Lakki Marwat 28420, Pakistan; asad@ulm.edu.pk

**Keywords:** molecular structure, chemical graphs, power graphs, finite groups, Hosoya index, Hosoya polynomial

## Abstract

Suppose G is a finite group. The power graph represented by P(G) of G is a graph, whose node set is G, and two different elements are adjacent if and only if one is an integral power of the other. The Hosoya polynomial contains much information regarding graph invariants depending on the distance. In this article, we discuss the Hosoya characteristics (the Hosoya polynomial and its reciprocal) of the power graph related to an algebraic structure formed by the symmetries of regular molecular gones. As a consequence, we determined the Hosoya index of the power graphs of the dihedral and the generalized groups. This information is useful in determining the renowned chemical descriptors depending on the distance. The total number of matchings in a graph Γ is known as the *Z*-index or Hosoya index. The *Z*-index is a well-known type of topological index, which is popular in combinatorial chemistry and can be used to deal with a variety of chemical characteristics in molecular structures.

## 1. Introduction

A topological index is a numeric value that represents the symmetry of a molecular structure. Indeed, it is a mathematical classification of a chemical graph that offers a mathematical function in a quantitative structure–property relationship (QSPR). It links numerous physicochemical characteristics of molecular structured chemical substances, such as the strain energy, stability, and boiling point. Numerous characteristics of chemical compounds having a molecular structure can be examined using several kinds of topological indices. In 1947, H. Wiener presented the notion of the first topological index in researching the boiling point of paraffin, which he called the path number [1]. As a result, it was dubbed the Wiener index, and it was the moment that the idea of topological indices began.

Polya’s [2] concept of computing the polynomials was used by many chemists to identify the molecular orbitals of the unsaturated hydrocarbons. In this context, the spectrum of a graph has been extensively examined. According to [3], Hosoya used this idea in 1988 to determine the polynomials of several chemical structures, which were labeled the Hosoya polynomials and attracted widespread attention. In 1996, Sagan et al. [4] then retitled the Hosoya polynomial as the Wiener polynomial, although most experts still refer to it as the Hosoya polynomial. The information regarding distance-based graph invariants may be obtained from the Hosoya polynomial. In [5], Cash noticed a connection between the hyper Wiener index and the Hosoya polynomial. Estrada et al. [6] investigated several interesting applications of the expanded Wiener indices.

The graphs presented in this paper are all simple graphs, meaning they have no loops nor multiple edges. Suppose G is a finite group. The power graph represented by P(G) of G is a graph, in which G is its node set and two unlike elements are edge connected if and only if one of them is an integer power of the other. In [7], Kelarev and Quinn discussed the approach of directed power graphs related to groups and semigroups. Later, the authors of [8] illustrated the power graph P(S) of a semigroup S and identified the class of semigroups, whose power graphs are the complete graphs. Furthermore, they discussed that for any finite group G, the associated power graph is the complete graph if and only if the group G is cyclic of order one or $p^{k}$, where *p* is any prime and k∈N.

In the current literature of theory of graphs, the power graph is now an exciting topic in several branches of mathematics, that is group theory, ring theory, and Lie algebra. Cameron et al. [9] discussed the matching numbers and gave the upper, as well as the lower bounds for the perfect matching of power graphs of certain finite groups. They also derived a formula of matching numbers for any finite nilpotent groups. The authors of [10,11,12,13] presented an overview of finite groups with enhanced power graphs that enable the formation of a perfect code. They further established all possible perfect codes of the proper reduced power graphs and gave a necessary and sufficient condition for graphs having perfect codes. In [14], the authors concentrated on the power indices graph and classified all such graphs in some specified categories.

The authors of [15] examined the maximum clique and found the largest number of edges of power graphs for all the finite cyclic groups. Sriparna et al. [16] deliberated about the node connectivity of $P(Z_{n})$, whenever *n* is the product of some prime numbers. Furthermore, several other researchers inquired about different concepts of algebraic graphs; for instance, see [17,18,19,20] and the references therein.

A matching or an independent edge set is the collection of edges that share no nodes. When a node is coincident with one of the matching edges, it is referred to as matched. Otherwise, there is an unmatched node. The maximum number of matchings in a graph is referred to as the *Z*-index or Hosoya index. Hosoya [21] first proposed the *Z*-index in 1971 and then extended the topological index as a common tool for quantum chemistry in [22]. It has since been shown to be useful in a variety of molecular chemistry problems, including the heat of vaporization, entropy, and the boiling point. The *Z*-index is a well-known topological index example that has significant relevance in combinatorial chemistry. Considering numerous graph structures, many researchers investigated extremal problems in regard to the *Z*-index. Extremal characteristics of different graphs, unicyclic graphs, and trees were extensively studied in [23,24,25,26].

In this article, we represent the cyclic group $Z_{n}$ of order *n*, the generalized quaternion group $Q_{4n}$, and the dihedral group $D_{2m}$ of order 4n and 2m, respectively. It is very challenging to calculate the (reciprocal) Hosoya polynomial, as well as the *Z*-index of power graph P(G) of a group G. In this regard, we provide both the Hosoya and the reciprocal Hosoya polynomials and also discuss the *Z*-index of the power graph P(G) of a group G, when G is $D_{2m}$ or $Q_{4n}$.

There are still several gaps in the current study about the determination of the Hosoya polynomials, the reciprocal Hosoya polynomials, and also the *Z*-index or Hosoya index of the power graphs of a finite cyclic group $Z_{n}$, the dihedral group $D_{2m}$, and the generalized quaternion group $Q_{4n}$. We look at one of these problems in this article.

## 2. Basic Notions and Notations

This part reviews several fundamental graph-theoretic properties and well-known findings that will be important later in the article.

Suppose Γ is a simple finite undirected graph. The node and edge sets of Γ are represented by V(Γ) and E(Γ), respectively. The distance from $u_{1}$ to $u_{2}$ in a connected graph Γ denoted by $dis(u_{1},\;u_{2})$ is defined as the shortest distance between $u_{1}$ and $u_{2}$. The total number of nodes, denoted by |Γ|, is said to be the order of Γ. Two nodes $v_{1}$ and $v_{2}$ are adjacent if there is an edge between them, and we denote them by $v_{1}∼v_{2},$ otherwise $v_{1}≁v_{2}.$ The valency or degree represented by $deg(u_{1})$ of a node $u_{1}$ is the collection of nodes in Γ, which are adjacent to $u_{1}$. A $u_{1}-u_{2}$ path having $dis(u_{1},u_{2})$ length is known as a $u_{1}-u_{2}$ geodesic. The largest distance between a node $u_{1}$ and any other node of Γ is known as the eccentricity and is denoted by $ec(u_{1})$. The diameter denoted by diam(Γ) of Γ is the largest eccentricity among all the nodes of the graph Γ. Furthermore, the radius symbolized by rad(Γ) of Γ is the lowest eccentricity among all the nodes of the graph Γ.

Suppose Γ is a graph of order *n*. According to Hosoya, the polynomial of Γ with a variable *y* is defined as follows:

$$H\left(\Gamma ,y\right)=\sum_{i\geq 0}dis\left(\Gamma ,i\right)y^{i}.$$

The coefficient dis(Γ,i) represents the number of pairs of nodes (v,w) so that dis(v,w)=i, where i≤diam(Γ). Ramane and Talwar [27] proposed the reciprocal status Hosoya polynomial of Γ, which is given as:

$$H_{rs}\left(\Gamma ,y\right)=\sum_{vw\in E(\Gamma )}y^{rs(v)+rs(w)},$$

where $rs\left(w\right)=\sum_{v\in V(\Gamma ),w\neq v}\frac{1}{dis(w,v)}$ is referred to as the transmission or the reciprocal status of a node *w*.

Suppose $\Gamma_{1}$ and $\Gamma_{2}$ are two connected graphs, then $\Gamma_{1}\vee \Gamma_{2}$ is the join of $\Gamma_{1}$ and $\Gamma_{2}$ whose node and edge sets are $V\left(\Gamma_{1}\right)\cup V\left(\Gamma_{2}\right)$ and $E\left(\Gamma_{1}\right)\cup E\left(\Gamma_{2}\right)\cup \left\{y∼z:y\in V\left(\Gamma_{1}\right),\;z\in V\left(\Gamma_{2}\right)\right\}$, respectively. A complete graph is a graph that has an edge between any single node in the graph, and it is symbolized by $K_{n}$. Other unexplained terminologies and notations were taken from [28].

**Definition** **1.** *Assume that G is a group. Then, the center of G is given as:*

$$Z\left(G\right)=\left\{g_{1}:g_{1}\in G\;\mathrm{and}\;g_{1}g_{2}=g_{2}g_{1},\;\mathrm{for}\;\mathrm{all}\;g_{2}\in G\right\}.$$

The dihedral group $D_{2m}$ is the group of symmetries, and its order is 2m, where m≥3. The presentation of a dihedral group is given by:

$$D_{2m}=\langle a,\;b:\;a^{m}=b^{2}=1,\;bab=a^{m-1}\rangle .$$

Throughout this paper, we mean $m=p^{\alpha },$ where α∈N and *p* is any odd prime number. We now split $D_{2m}$ as follows:

$$H_{1}=\langle a\rangle ,H_{2}=\overset{\frac{m}{2}-1}{\underset{ı=0}{⋃}}H_{2}^{ı}=\left\{a^{ı}b,\;a^{\frac{m}{2}+ı}b\right\}=\left\{b,\;ab,\;a^{2}b,\ldots ,\;a^{m-1}b\right\},$$

where $0\leq ı\leq \frac{m}{2}-1$ and $H_{3}=\langle a\rangle \backslash \left\{e\right\}$. Since |a|=m and $|a^{i}b|=2$, for all 1≤i≤m, where the identity *e* is connected to every other node in its power graph, the subgraph induced by $H_{1}$ is a complete graph $K_{m}$.

Furthermore, the presentation of generalized quaternion group $Q_{4n}$ of order 4n for $n=2^{k}$, where k∈N, is given as:

$$Q_{4n}=\langle y,z\;|\;y^{2n}=1,\;y^{n}=z^{2},\;zyz^{-1}=y^{-1}\rangle .$$

We now split $Q_{4n}$ as follows:

$$\Omega =\left\{e,y^{n}\right\},\;A_{1}=\langle y\rangle ,\;A_{2}=\overset{n-1}{\underset{i=0}{⋃}}A_{2}^{i},\;\mathrm{where}\;A_{2}^{i}=\left\{y^{i}z,\;y^{n+i}z\right\}$$

and $A_{3}=A_{1}\backslash \Omega$. Since $A_{1}$ is cyclic, its induced subgraph is complete, and it is denoted by $K_{2n}$. A remarkable feature of $Q_{4n}$ is that the involution $y^{n}$ and the identity *e* are adjacent to every other node in their power graph. Several researchers [29,30,31,32] have analyzed the complete description of the above-mentioned groups and their power graphs. The first survey paper on power graphs was published in 2013 [33], and the most recent study was [34].

**Proposition** **1** ([31])**.** *The power graph $P(D_{2m})$ of $D_{2m}$ satisfies:*

$$P\left(D_{2m}\right)=K_{1}\vee \left(K_{m-1}\cup {\bar{K}}_{m}\right).$$

**Proposition** **2** ([31])**.** *The power graph $P(Q_{4n})$ of $Q_{4n}$ satisfies:*

$$P\left(Q_{4n}\right)=K_{2}\vee \left(K_{2n-2}\cup nK_{2}\right),$$

*where $nK_{2}$ represents the n copies of $K_{2}$.*

## 3. Hosoya Properties

The Hosoya polynomial and its reciprocal status of the power graphs of the dihedral and generalized quaternion groups are determined in this section.

### 3.1. Main Results

**Theorem** **1.** *Let $P\left(D_{2m}\right)$ be the power graph of $D_{2m}.$ Then:*

$$H_{rs}\left(P(D_{2m})\right)=\left(m-1\right)y^{\frac{7m-4}{2}}+my^{3m-1}+\left(\frac{m-1}{2}\right)y^{3m-2}.$$

**Theorem** **2.** *Suppose $P(Q_{4n})$ is a power graph of $Q_{4n}.$ Then:*

$$H_{rs}\left(P(Q_{4n})\right)=\left(\frac{2(n-1)}{2}\right)y^{2(3n-1)}+4\left(n-1\right)y^{7n-2}+y^{2(4n-1)}+4ny^{6n}+ny^{2(2n+1)}.$$

**Theorem** **3.** *The Hosoya index of $P(D_{2m})$ is as follows:*

$$1+\left(\frac{m}{2}\right)+m+\sum_{i=2}^{\frac{m}{2}}\left[\left(\frac{1}{i}\right)\prod_{k=0}^{i-1}\left(\frac{m-2k}{2}\right)+\left(\frac{m}{i-1}\right)\prod_{k=0}^{i-2}\left(\frac{m-2k-1}{2}\right)\right].$$

**Theorem** **4.** *For n≥2, the Hosoya index of $P(Q_{4n})$ is given as:*

$$1+\sum_{i=1}^{n}d_{i}^{1}+\sum_{i=1}^{2}d_{i}^{2}+\sum_{i=1}^{n}d_{i}^{3}+\sum_{i=2}^{n+1}d_{i}^{4}+d_{2}^{5}+\sum_{i=2}^{n}d_{i}^{6}+\sum_{i=2}^{2n}d_{i}^{7},$$

*where:*

$$\begin{array}{ll} d_{i}^{1} & =\frac{1}{i}\prod_{k=0}^{i-1}\left(\frac{2(n-k)}{2}\right),\;d_{1}^{2}=4n,\;d_{2}^{2}=4n\left(n-\frac{1}{2}\right),\\ d_{i}^{3} & =\left(\frac{n}{i}\right),\;d_{2}^{4}=4n\left(\frac{2(n-1)}{2}\right),\\ d_{i}^{4} & =2n\left\{\frac{2}{i-1}\prod_{k=0}^{i-2}\left(\frac{2(n-k-1)}{2}\right)+\frac{2n-1}{i-2}\prod_{k=0}^{i-3}\left(\frac{2(n-k-1)}{2}\right)\right\}, \end{array}$$

*where 3≤i≤n,*

$$\begin{array}{ll} d_{n+1}^{4} & =\frac{4n(n-\frac{1}{2})}{i-2}\prod_{k=0}^{i-3}\left(\frac{2(n-k-1)}{2}\right),d_{2}^{5}=8n\left(n-\frac{1}{2}\right),d_{2}^{6}=4n\left(n-1\right),\\ d_{i}^{6} & =2n\left\{2\left(\frac{n-1}{i-1}\right)+\left(\frac{n-1}{i-2}\right)+4\left(n-1\right)\left(\frac{n-2}{i-2}\right)\right\},\;\mathrm{where}\;3\leq i\leq n,\\ d_{i}^{7} & =\sum_{j=1}^{i-1}\frac{1}{j}\prod_{k=0}^{j-1}\left(\frac{2(n-k)}{2}\right)\left(\frac{n}{i-j}\right),\;\mathrm{where}\;2\leq i\leq 2n. \end{array}$$

### 3.2. Hosoya Polynomial

To find the Hosoya polynomial, we first prove some important results.

**Proposition** **3.** *Suppose $P(D_{2m})$ is the power graph of $D_{2m}.$ Then:*

$$dis\left(P(D_{2m}),\ell \right)=\left\{\begin{array}{cc} 2m, & \;\mathrm{for}\ell =0;\\ \frac{m(m+1)}{2}, & \;\mathrm{for}\ell =1;\\ \frac{3m(m-1)}{2}, & \;\mathrm{for}\ell =2. \end{array}\right.$$

**Proof.**  As we know that $diam(P\left(D_{2m}\right))=2$, we need to determine $dis\left(P(D_{2m}),\;0\right),$ $dis\left(P(D_{2m}),\;1\right)$, and $dis\left(P(D_{2m}),\;2\right)$. Now, take a node set $V_{k}$ for any pair of nodes of $P(D_{2m}),$ then:

$$|V_{k}|=\left(\frac{\left|P(D_{2m})\right|}{2}\right)+\left|P\left(D_{2m}\right)\right|=m\left(2m+1\right).$$

Suppose:

$$C\left(P\left(D_{2m}\right),\ell \right)=\left\{\left(i,j\right);i,j\in V\left(P\left(D_{2m}\right)\right)\;|\;dis\left(i,j\right)=\ell \right\},$$

and $dis\left(P\left(D_{2m}\right),\ell \right)=\left|C\left(P\left(D_{2m}\right),\ell \right)\right|.$ Then:

(1) $$V_{k}=C\left(P\left(D_{2m}\right),0\right)\cup C\left(P\left(D_{2m}\right),1\right)\cup C\left(P\left(D_{2m}\right),2\right).$$

As we know, dis(i,i)=0,∀$i\in V(P\left(D_{2m}\right))$, so:

$$C\left(P\left(D_{2m}\right),0\right)=\left\{\left(i,i\right);i\in V(P\left(D_{2m}\right))\right\},$$

and this is actually $V(P\left(D_{2m}\right)).$ Thus, $C(P\left(D_{2m}\right),0)=2m.$ Using Proposition 1, $P\left(D_{2m}\right)=K_{1}\vee \left(K_{m-2}\cup \bar{K_{m}}\right)$ with $V\left(K_{1}\right),V\left(K_{m-1}\right)=H_{3},$ and $V\left(K_{m}\right)=H_{2}$. Therefore,

$$\begin{array}{cc} C(P\left(D_{2m}\right),1) & =\left\{\left(i,j\right);i=e,j\in H_{2}\right\}\cup \left\{\left(i,j\right);i=e,j\in H_{3}\right\}\\ & \cup \left\{\left(i,j\right);i,j\in H_{3}\;\mathrm{and}\;i\neq j\right\}. \end{array}$$

Consequently, $C\left(P\left(D_{2m}\right),1\right)=m+\left(m-1\right)+\left(\frac{m-1}{2}\right)=\frac{m(m+1)}{2}$. Using Equation (1), we obtain:

$$|V_{k}|=dis\left(P(D_{2m}),0\right)+dis\left(P(D_{2m}),1\right)+dis\left(P(D_{2m}),2\right).$$

Hence,

$$\begin{array}{cc} dis\left(P(D_{2m}),2\right) & =|V_{k}|-dis\left(P(D_{2m}),0\right)-dis\left(P(D_{2m}),1\right)\\ & =m\left(2m+1\right)-2m-\frac{m(m+1)}{2}\\ & =\frac{3m(m-1)}{2}. \end{array}$$

□

**Proposition** **4.** *Suppose $P(Q_{4n})$ is the power graph of $Q_{4n}.$ Then:*

$$dis\left(P(Q_{4n}),\ell \right)=\left\{\begin{array}{cc} 4n, & \;\mathrm{for}\;\ell =0;\\ 2n(n+2), & \;\mathrm{for}\;\ell =1;\\ 6n(n-1), & \;\mathrm{for}\;\ell =2. \end{array}\right.$$

**Proof.**  As we know that $diam(P\left(Q_{4n}\right))=2$, we need to determine $dis\left(P(Q_{4n}),\;0\right)$, $dis\left(P(Q_{4n}),\;1\right)$, and $dis\left(P(Q_{4n}),\;2\right)$. Suppose $V_{k}$ is the collection of all pairs of nodes of $P(Q_{4n}),$ then:

$$|V_{k}|=2n\left(4n+1\right).$$

Let

$$C\left(P\left(Q_{4n}\right),\ell \right)=\left\{\left(i,j\right);i,j\in V\left(P\left(Q_{4n}\right)\right)|dis\left(i,j\right)=\ell \right\},$$

then $dis\left(P\left(Q_{4n}\right),\ell \right)=\left|C\left(P\left(Q_{4n}\right),\ell \right)\right|,$ and:

(2) $$V_{k}=C\left(P\left(Q_{4n}\right),0\right)\cup C\left(P\left(Q_{4n}\right),1\right)\cup C\left(P\left(Q_{4n}\right),2\right).$$

Since, dis(i,i)=0, for any $i\in V(P\left(Q_{4n}\right))$, so:

$$C\left(P\left(Q_{4n}\right),0\right)=\left\{\left(i,i\right);i\in V(P\left(Q_{4n}\right))\right\}$$

and this is equal to $V(P\left(Q_{4n}\right)).$ Thus, $C(P\left(Q_{4n}\right),0)=4n.$ Using Proposition 2, where $V\left(K_{2}\right)=\left\{e,y^{n}\right\},$$V\left(K_{2n-2}\right)=A_{3},$ and $V\left(nK_{n}\right)=A_{2}=\overset{n-1}{\underset{j=0}{⋃}}A_{2}^{j}$, therefore, we have:

$$\begin{array}{cc} C(P\left(Q_{4n}\right),1) & =\left\{\left(i,j\right);i\in \Omega ,j\in A_{2}\right\}\cup \overset{n-1}{\underset{j=0}{⋃}}\left\{\left(i,j\right);\left(i,j\right)\in A_{2}^{j}\;\mathrm{and}\;j\neq i\right\}\\ & \cup \left\{\left(i,j\right);i\in \Omega ,j\in A_{3}\right\}\cup \left\{\left(i,j\right);\left(i,j\right)\in A_{3}\;\mathrm{and}\;j\neq i\right\}\\ & \cup \left\{(i,j);(i,j)\in \Omega \;\mathrm{and}\;j\neq i\right\}. \end{array}$$

Consequently, $dis\left(P\left(Q_{4n}\right),1\right)=4n+n\left(1\right)+2\left(2n-2\right)+\left(\frac{2n-2}{2}\right)+1=2n\left(n+2\right)$. Using Equation (2), we obtain:

$$|V_{k}|=dis\left(P(Q_{4n}),0\right)+dis\left(P(Q_{4n}),1\right)+dis\left(P(Q_{4n}),2\right).$$

Hence,

$$\begin{array}{cc} dis\left(P(Q_{4n}),2\right) & =|V_{k}|-dis\left(P(Q_{4n}),0\right)-dis\left(P(Q_{4n}),1\right)\\ & =2n(4n+1)-4n-2n(n+2)\\ & =6n(n-1). \end{array}$$

□

The following results yield the Hosoya polynomials of the power graphs of the dihedral and the generalized quaternion groups.

**Theorem** **5.** *Consider the power graph $P(D_{2m})$ of $D_{2m}$. Then:*

$$H\left(P\left(D_{2m}\right),y\right)=\frac{m}{2}\left(3\left(m-1\right)y^{2}+\left(m+1\right)y+4\right).$$

**Proof.**  By substituting the coefficients $dis(P\left(D_{2m}\right),\ell )$ derived in Propositions 3 and 4 into the formula for the Hosoya polynomial, we obtain:

$$\begin{array}{cc} H(P\left(D_{2m}\right),y) & =dis\left(P(D_{2m}),2\right)y^{2}+dis\left(P(D_{2m}),1\right)y^{1}+dis\left(P(D_{2m}),0\right)y^{0}\\ & =\left(\frac{3m(m-1)}{2}\right)y^{2}+\left(\frac{m(m+1)}{2}\right)y+2my^{0}\\ & =\frac{m}{2}\left(3\left(m-1\right)y^{2}+\left(m+1\right)y+4\right). \end{array}$$

We obtain the desired polynomial. □

**Theorem** **6.** *Consider the power graph $P(Q_{4n})$ of $Q_{4n}$. Then:*

$$H\left(P\left(Q_{4n}\right),y\right)=n\left(6\left(n-1\right)y^{2}+2\left(n+2\right)y+4\right).$$

**Proof.**  By substituting the coefficients $dis(P\left(Q_{4n}\right),\ell )$ derived in Propositions 3 and 4 into the formula for the Hosoya polynomial, we obtain:

$$\begin{array}{cc} H(P\left(Q_{4n}\right),y) & =dis\left(P(Q_{4n}),2\right)y^{2}+dis\left(P(Q_{4n}),1\right)y^{1}+dis\left(P(Q_{4n}),0\right)y^{0}\\ & =\left(6n(n-1)\right)y^{2}+\left(2n(n+2)\right)y+\left(4n\right)y^{0}\\ & =n\left(6\left(n-1\right)y^{2}+2\left(n+2\right)y+4\right). \end{array}$$

This proves the statement. □

## 4. Reciprocal Status Hosoya Polynomial

This part determines the reciprocal status of every node in the power graphs.

**Proposition** **5.** *If v is a node of $P(D_{2m})$, then:*

$$rs\left(v\right)=\left\{\begin{array}{cc} 2m-1, & \;\mathrm{when}\;v=e;\\ m, & \;\mathrm{when}\;v\in H_{2};\\ \frac{3m-2}{2}, & \;\mathrm{when}\;v\in H_{3}. \end{array}\right.$$

**Proof.**  Using Proposition 1, $P\left(D_{2m}\right)=K_{1}\vee \left(K_{m-1}\cup {\bar{K}}_{m}\right)$ with node set $\left\{e\right\}\cup H_{2}\cup H_{3}$. Therefore, if v=e, then ec(v)=1, and following the concept of the reciprocal status, we have:

$$rs\left(v\right)=\left(\frac{1}{1}\right)\left(m+m-1\right)=2m-1.$$

When $v\in H_{2}$, implying ec(v)=2, also, we apply the concept of the reciprocal status, so we have:

$$rs\left(v\right)=\left(\frac{1}{1}\right)\left(1\right)+\frac{1}{2}\left(2m-2\right)=m.$$

When $v\in H_{3}$, implying ec(v)=2, also, we apply the concept of the reciprocal status, so we have:

$$rs\left(v\right)=\left(\frac{1}{1}\right)\left(m-1\right)+\frac{m}{2}=\frac{3m-2}{2}.$$

Combining them, we obtain the required quantity. □

**Proposition** **6.** *If v is a node of $P(Q_{4n})$, then:*

$$rs\left(v\right)=\left\{\begin{array}{cc} 4n-1, & \;\mathrm{when}\;v\in \Omega \\ 2n+1, & \;\mathrm{when}\;v\in A_{2}\\ 3n-1, & \;\mathrm{when}\;v\in A_{3} \end{array}\right.$$

**Proof.**  Using Proposition 2, the node set of $P(Q_{4n})$ is $\Omega \cup A_{2}\cup A_{3}$. Therefore, when v∈Ω, implying ec(v)=1, also, we apply the concept of the reciprocal status, so we have:

$$rs\left(v\right)=\left(\frac{1}{1}\right)\left\{1+2n-2+2n\right\}=4n-1.$$

When $v\in A_{2}$, implying ec(v)=2, also, we apply the concept of the reciprocal status, so we have:

$$rs\left(v\right)=3\left(\frac{1}{1}\right)+\left(\frac{1}{2}\right)\left\{2\left(n-1\right)+\left(\frac{4n}{2}-2\right)\right\}=2n+1.$$

When $v\in A_{3}$, implying ec(v)=2, and by the definition of the reciprocal status, we have:

$$rs\left(v\right)=\left(\frac{1}{1}\right)\left(2n-3+2\right)+\left(\frac{1}{2}\right)\left(2n\right)=3n-1.$$

Combining them, we obtain the required quantity. □

### Proof of Theorems 1 and 2

The following results compute the reciprocal status Hosoya polynomial of the power graph of a group G.

**Proof of Theorem** **1.** Using Proposition 5, this means that there are three types of edges (u∼v,u∼w,v∼v) in $P(D_{2m})$, according to their end nodes’ reciprocal statuses, where $u=2m-1,v=\frac{3m-2}{2}$, and c=m. The edge partition is shown in the following Table 1.

By incorporating the edge set’s partition specified in Table 1 into the formula for the reciprocal status Hosoya polynomial, we obtain the following.

$$\begin{array}{cc} H_{rs}\left(P\left(D_{2m}\right)\right) & =\sum_{E_{u∼v}}y^{u+v}+\sum_{E_{u∼w}}y^{u+w}+\sum_{E_{v∼v}}y^{v+v}\\ & =\left(m-1\right)y^{2m-1+\frac{3m-2}{2}}+my^{2m-1+m}+\left(\frac{m-1}{2}\right)y^{\frac{3m-2}{2}+\frac{3m-2}{2}}\\ & =\left(m-1\right)y^{\frac{7m-4}{2}}+my^{3m-1}+\left(\frac{m-1}{2}\right)y^{3m-2}. \end{array}$$

□

**Table 1:** $P(D_{2m})$ is partitioned into edges based on their reciprocal statuses.

Type of Edge	Edge Set’s Partition	Edges Count
u∼v	$E_{u∼v}=\left\{ab\in E\left(P\left(D_{2m}\right)\right):rs\left(a\right)=u,rs\left(b\right)=v\right\}$	$|E_{u∼v}|\;=\;m-1$
u∼w	$E_{u∼w}=\left\{ab\in E\left(P\left(D_{2m}\right)\right):rs\left(a\right)=u,rs\left(b\right)=w\right\}$	$|E_{u∼w}|\;=\;m$
v∼v	$E_{v∼v}=\left\{ab\in E\left(P\left(D_{2m}\right)\right):rs\left(a\right)=v,rs\left(b\right)=v\right\}$	$|E_{v∼v}|\;=\;\left(\frac{m-1}{2}\right)$

**Proof of Theorem** **2.** From Proposition 6, there are five types of edges (u∼u,u∼v,v∼v,v∼w,w∼w) in $P(Q_{4n})$. Consequently, the edge partitioning is shown in Table 2 along with the reciprocal status of their end nodes, when $u=\frac{6n}{2}-1$, v=4n−1,w=2n+1.

We obtain the reciprocal status Hosoya polynomial formula by substituting the edge partition of $P(Q_{4n})$, which is provided in Table 2.

$$\begin{array}{cc} H_{rs}\left(P(Q_{4n})\right) & =\sum_{E_{u∼u}}y^{u+u}+\sum_{E_{u∼v}}y^{u+v}+\sum_{E_{v∼v}}y^{v+v}+\sum_{E_{v∼w}}y^{v+w}+\sum_{E_{w∼w}}y^{w+w}\\ & =\left(\frac{2(n-1)}{2}\right)y^{2(3n-1)}+4\left(n-1\right)y^{\left(3n-1)+(4n-1\right)}+\left(1\right)y^{2(4n-1)}\\ & +\left(4n\right)y^{\left(4n-1)+(2n+1\right)}+\left(n\right)y^{2(2n+1)}\\ & =\left(\frac{2(n-1)}{2}\right)y^{2(3n-1)}+4\left(n-1\right)y^{7n-2}+y^{2(4n-1)}+\left(4n\right)y^{6n}+\left(n\right)y^{2(2n+1)}. \end{array}$$

□

**Table 2:** $P(Q_{4n})$ is partitioned into edges based on their reciprocal statuses.

Type of Edge	Edge Set’s Partition	Edges Count
u∼u	$E_{u∼u}=\left\{ab\in E\left(P\left(D_{2m}\right)\right):rs\left(a\right)=u,rs\left(b\right)=u\right\}$	$|E_{u∼u}|\;=\;\left(\frac{2(n-1)}{2}\right)$
u∼v	$E_{u∼v}=\left\{ab\in E\left(P\left(D_{2m}\right)\right):rs\left(a\right)=u,rs\left(b\right)=v\right\}$	$|E_{u∼v}|\;=\;4\left(n-1\right)$
v∼v	$E_{v∼v}=\left\{ab\in E\left(P\left(D_{2m}\right)\right):rs\left(a\right)=v,rs\left(b\right)=v\right\}$	$|E_{v∼v}|\;=\;1$
v∼w	$E_{v∼w}=\left\{ab\in E\left(P\left(D_{2m}\right)\right):rs\left(a\right)=v,rs\left(b\right)=w\right\}$	$|E_{v∼w}|\;=\;4n$
w∼w	$E_{w∼w}=\left\{ab\in E\left(P\left(D_{2m}\right)\right):rs\left(a\right)=w,rs\left(b\right)=w\right\}$	$|E_{w∼w}|\;=\;n$

## 5. Hosoya Index

The Hosoya index of the power graphs of finite groups is examined in this section. On a graph with *n* nodes, the greatest feasible value of the Hosoya index is provided by the complete graph $K_{n}$ [35]. In general, the Hosoya index of $K_{n},$ where n≥2, is as follows:

$$1+\sum_{i=1}^{\frac{n}{2}}\left(\frac{1}{i}\right)\prod_{k=0}^{i-1}\left(\frac{n-2k}{2}\right),$$

this may be seen concerning the entire non-void matchings specified in Table 3, whereas $d_{i}$ represents the cardinality *i* matchings, where $1\leq i\leq \frac{n}{2}.$

**Table 3:** The total non-void matchings in $K_{n}$.

$K_{m}$	$d_{1}$	$d_{2}$	$d_{3}$	$d_{4}$	⋯	$d_{i}$
$K_{2}$	$\left(\frac{2}{2}\right)$					
$K_{3}$	$\left(\frac{3}{2}\right)$					
$K_{4}$	$\left(\frac{4}{2}\right)$	$\frac{1}{2}\left(\frac{4}{2}\right)\left(\frac{2}{2}\right)$				
$K_{5}$	$\left(\frac{5}{2}\right)$	$\frac{1}{2}\left(\frac{5}{2}\right)\left(\frac{3}{2}\right)$				
$K_{6}$	$\left(\frac{6}{2}\right)$	$\frac{1}{2}\left(\frac{6}{2}\right)\left(\frac{4}{2}\right)$	$\frac{1}{3}\left(\frac{6}{2}\right)\left(\frac{4}{2}\right)\left(\frac{2}{2}\right)$			
$K_{7}$	$\left(\frac{7}{2}\right)$	$\frac{1}{2}\left(\frac{7}{2}\right)\left(\frac{5}{2}\right)$	$\frac{1}{3}\left(\frac{7}{2}\right)\left(\frac{5}{2}\right)\left(\frac{3}{2}\right)$			
$K_{8}$	$\left(\frac{8}{2}\right)$	$\frac{1}{2}\left(\frac{8}{2}\right)\left(\frac{6}{2}\right)$	$\frac{1}{3}\left(\frac{8}{2}\right)\left(\frac{6}{2}\right)\left(\frac{4}{2}\right)$	$\frac{1}{4}\left(\frac{8}{2}\right)\left(\frac{6}{2}\right)\left(\frac{4}{2}\right)\left(\frac{2}{2}\right)$		
⋮	⋮	⋮	⋮	⋮	⋱	⋮
$K_{m}$	$\left(\frac{m}{2}\right)$	$\frac{1}{2}\left(\frac{m}{2}\right)\left(\frac{m-2}{2}\right)$	$\frac{1}{3}\left(\frac{m}{2}\right)\left(\frac{m-2}{2}\right)\left(\frac{m-4}{2}\right)$	$\frac{1}{4}\left(\frac{m}{2}\right)\left(\frac{m-2}{2}\right)\left(\frac{m-4}{2}\right)\left(\frac{m-6}{2}\right)$	⋯	$\frac{1}{i}\prod_{k=0}^{i-1}\left(\frac{m-2k}{2}\right)$

**Proof of Theorem** **3.** From the structure of $P(D_{2m})$, the identity *e* is the only node that is connected to every other node in $V\left(P(D_{2m})\right)$. Therefore, there are three types of edges in $P(D_{2m})$, i.e.,:

**Type** **1:** $u_{1}∼u_{2}$, for $u_{1},u_{2}\in H_{3};$
**Type** **2:** $u_{1}∼u_{2}$, for $u_{1}\in H_{3},u_{2}=e;$
**Type** **3:** $u_{1}∼u_{2}$, for $u_{1}\in H_{2},u_{2}=e.$

Since we know that the subgraph induced by $H_{1}$ is complete, i.e., $K_{m}$, thus, $P(D_{2m})$ has two distinct types of matchings:

$T_{1}\;\;\mathrm{Matchings}\;\mathrm{of}\;\mathrm{Type}\;1\;\mathrm{and}\;\mathrm{Type}\;2\;\mathrm{edges};$

$T_{2}\;\;\mathrm{Matchings}\;\mathrm{of}\;\mathrm{Type}\;1\;\mathrm{and}\;\mathrm{Type}\;3\;\mathrm{edges}:$

*T*_1_: For each type, the number of matchings may be calculated as follows: Due to the fact that the edges of Type 1 and Type 2 are the edges of a complete graph $K_{m}$, which is induced by the nodes in $H_{1}$, so the number of matchings in this type can be obtained by counting the matchings in $K_{m}$, which are given in Table 4, where $d_{i}$ denotes the number of matchings of order *i*, for $1\leq i\leq \frac{m}{2};$
*T*_2_: Every matching of this kind may be generated by substituting one edge of Type 3 for any edge of Type 1. Given that every Type-1 edge is also an edge of $K_{m-1}$, which is induced by the nodes of $H_{3}$, so every matching of Type-1 edges is also a matching of $K_{m-1}$. The total number of certain matchings is described in Table 5, where for any $1\leq i\leq \frac{m}{2}$, $d_{i}$ signifies the total number of matchings of order *i*.

Given that Type 3 has *m* edges, the essential matchings may be produced as:

  - Matchings having one order: These are the *m* such matches that correspond to the *m* Type-3 edges;
  - Matchings having two orders: All of these matchings may be achieved by inserting a Type-3 edge through every other matching having one order in $K_{m-1}$. Using Table 5, there are *m* edges of Type 3, as well as $\left(\frac{m-1}{2}\right)$ matchings having one order in $K_{m-1}$. As a result of the product rule, the number of matchings having two order is: $m\left(\frac{m-1}{2}\right)$;
  - Matchings having three orders: Every one of these matchings may be achieved by inserting a Type-3 edge into every other matching of order two in $K_{m-1}$. Thus, there are *m* Type-3 edges, as well as $\frac{1}{2}\left(\frac{m-1}{2}\right)\left(\frac{m-3}{2}\right)$ matchings having two order in $K_{m-1}$, by Table 5. As a result of the product rule, the number of order three matchings is:
    $$\frac{m}{2}\left(\frac{m-1}{2}\right)\left(\frac{m-3}{2}\right);$$
  - Order four matchings: Any of these matchings may be generated by inserting one edge of Type 3 through every order three matching in $K_{m-1}$. Thus, there are *m* Type-3 edges, as well as $\frac{1}{3}\left(\frac{m-1}{2}\right)\left(\frac{m-3}{2}\right)\left(\frac{m-5}{2}\right)$ matchings having three orders in $K_{m-1}$, by Table 5. As a consequence of the product rule, the number of matchings of order four is given by:
    $$\frac{m}{3}\left(\frac{m-1}{2}\right)\left(\frac{m-3}{2}\right)\left(\frac{m-5}{2}\right);$$
  - Order *i* matchings: In general, every order *i* matching may be generated by adding one edge of Type 3 to every order i−1 matching in $K_{m-1}$. According to Table 5, there are *m* possible edges of Type 3 and $\frac{1}{i-1}\prod_{k=0}^{i-2}\left(\frac{m-2k-1}{2}\right)$ matchings of order i−1 in $K_{m-1}$. Therefore, by the product rule, the number of matchings of order *i* is:
    $$\frac{m}{i-1}\prod_{k=0}^{i-2}\left(\frac{m-2k-1}{2}\right).$$

Next, using the sum rule, the entire matchings in each order (matchings $\left(T_{1}\right)$ + matchings $\left(T_{2}\right)$) may be calculated as: The number of matchings of order one is as follows:

$$m+\left(\frac{m}{2}\right).$$

Order two has the sequel number of matchings:

$$m\left(\frac{m-1}{2}\right)+\frac{1}{2}\left(\frac{m}{2}\right)\left(\frac{m-2}{2}\right).$$

Order three has the following number of matchings:

$$\frac{m}{2}\left(\frac{m-1}{2}\right)\left(\frac{m-3}{2}\right)+\frac{1}{3}\left(\frac{m}{2}\right)\left(\frac{m-2}{2}\right)\left(\frac{m-4}{2}\right).$$

Order four has the following number of matchings:

$$\frac{m}{3}\left(\frac{m-1}{2}\right)\left(\frac{m-3}{2}\right)\left(\frac{m-5}{2}\right)+\frac{1}{4}\left(\frac{m}{2}\right)\left(\frac{m-2}{2}\right)\left(\frac{m-4}{2}\right)\left(\frac{m-6}{2}\right).$$

Generally, order *i* has the following number of matchings:

$$\frac{1}{i}\prod_{k=0}^{i-1}\left(\frac{m-2k}{2}\right)+\frac{m}{i-1}\prod_{k=0}^{i-2}\left(\frac{m-2k-1}{2}\right),$$

where $2\leq i\leq \frac{m}{2}.$

Thus, the Hosoya index of $P(D_{2m})$ is given by:

$$1+m+\left(\frac{m}{2}\right)+\sum_{i=2}^{\frac{m}{2}}\left\{\frac{1}{i}\prod_{k=0}^{i-1}\left(\frac{m-2k}{2}\right)+\frac{m}{i-1}\prod_{k=0}^{i-2}\left(\frac{m-2k-1}{2}\right)\right\}.$$

□

**Table 4:** The total non-void matchings in $K_{m}$.

$K_{m}$	$d_{1}$	$d_{2}$	$d_{3}$	$d_{4}$	⋯	$d_{i}$
$K_{3}$	$\left(\frac{3}{2}\right)$					
$K_{5}$	$\left(\frac{5}{2}\right)$	$\frac{1}{2}\left(\frac{5}{2}\right)\left(\frac{3}{2}\right)$				
$K_{7}$	$\left(\frac{7}{2}\right)$	$\frac{1}{2}\left(\frac{7}{2}\right)\left(\frac{5}{2}\right)$	$\frac{1}{3}\left(\frac{7}{2}\right)\left(\frac{5}{2}\right)\left(\frac{3}{2}\right)$			
$K_{9}$	$\left(\frac{9}{2}\right)$	$\frac{1}{2}\left(\frac{9}{2}\right)\left(\frac{7}{2}\right)$	$\frac{1}{3}\left(\frac{9}{2}\right)\left(\frac{7}{2}\right)\left(\frac{5}{2}\right)$	$\frac{1}{4}\left(\frac{9}{2}\right)\left(\frac{7}{2}\right)\left(\frac{5}{2}\right)\left(\frac{3}{2}\right)$		
⋮	⋮	⋮	⋮	⋮	⋱	⋮
$K_{m}$	$\left(\frac{m}{2}\right)$	$\frac{1}{2}\left(\frac{m}{2}\right)\left(\frac{m-2}{2}\right)$	$\frac{1}{3}\left(\frac{m}{2}\right)\left(\frac{m-2}{2}\right)\left(\frac{m-4}{2}\right)$	$\frac{1}{4}\left(\frac{m}{2}\right)\left(\frac{m-2}{2}\right)\left(\frac{m-4}{2}\right)\left(\frac{m-6}{2}\right)$	⋯	$\frac{1}{i}\prod_{k=0}^{i-1}\left(\frac{m-2k}{2}\right)$

**Table 5:** The total non-void matchings in $K_{m}$.

$K_{m}$	$d_{1}$	$d_{2}$	$d_{3}$	$d_{4}$	⋯	$d_{i}$
$K_{2}$	$\left(\frac{2}{2}\right)$					
$K_{4}$	$\left(\frac{4}{2}\right)$	$\frac{1}{2}\left(\frac{4}{2}\right)\left(\frac{2}{2}\right)$				
$K_{6}$	$\left(\frac{6}{2}\right)$	$\frac{1}{2}\left(\frac{6}{2}\right)\left(\frac{4}{2}\right)$	$\frac{1}{3}\left(\frac{6}{2}\right)\left(\frac{4}{2}\right)\left(\frac{2}{2}\right)$			
$K_{8}$	$\left(\frac{8}{2}\right)$	$\frac{1}{2}\left(\frac{8}{2}\right)\left(\frac{6}{2}\right)$	$\frac{1}{3}\left(\frac{8}{2}\right)\left(\frac{6}{2}\right)\left(\frac{4}{2}\right)$	$\frac{1}{4}\left(\frac{8}{2}\right)\left(\frac{6}{2}\right)\left(\frac{4}{2}\right)\left(\frac{2}{2}\right)$		
⋮	⋮	⋮	⋮	⋮	⋱	⋮
$K_{m-1}$	$\left(\frac{m-1}{2}\right)$	$\frac{1}{2}\left(\frac{m-1}{2}\right)\left(\frac{m-3}{2}\right)$	$\frac{1}{3}\left(\frac{m-1}{2}\right)\left(\frac{m-3}{2}\right)\left(\frac{m-5}{2}\right)$	$\frac{1}{4}\left(\frac{m-1}{2}\right)\left(\frac{m-3}{2}\right)\left(\frac{m-5}{2}\right)\left(\frac{m-7}{2}\right)$	⋯	$\frac{1}{i-1}\prod_{k=0}^{i-2}\left(\frac{m-2k-1}{2}\right)$

**Proof of Theorem** **4.** Using Proposition 2, the node set is $V\left(P(Q_{4n})\right)=\Omega \cup A_{2}\cup A_{3}$, where $A_{2}={⋃}_{j=0}^{n-1}A_{2}^{j}$. Therefore, we have the sequel types of edges in $P(Q_{4n})$:

**Type** **1:** $u_{1}∼u_{2}$, for any $u_{1},u_{2}\in A_{3};$
**Type** **2:** $u_{1}∼u_{2}$, for any $u_{1},u_{2}\in \Omega ;$
**Type** **3:** $u_{1}∼u_{2}$, for any $u_{1}\in A_{3},u_{2}\in \Omega ;$
**Type** **4:** $u_{1}∼u_{2}$, for any $u_{1}\in A_{2},u_{2}\in \Omega ;$
**Type** **5:** $u_{1}∼u_{2}$, for any $u_{1},u_{2}\in A_{2}^{j}\subseteq A_{2},$ where 0≤j≤n−1.

Seven cases of matchings occur amongst the edges of $P(Q_{4n})$ according to the following categories:

$\left(d^{1}\right)$ Matchings amongst the Type-1, Type-2, as well as Type-3 edges;
$\left(d^{2}\right)$ Matchings amongst the Type-4 edges;
$\left(d^{3}\right)$ Matchings amongst the Type-5 edges;
$\left(d^{4}\right)$ Matchings amongst the Type-1 and Type-4 edges;
$\left(d^{5}\right)$ Matchings amongst the Type-3 and Type-4 edges;
$\left(d^{6}\right)$ Matchings amongst the Type-4 and Type-5 edges;
$\left(d^{7}\right)$ Matchings amongst the Type-1, Type-2, Type-3, and Type-5 edges.

The following method computes all the types of matchings as mentioned above:

$\left(d^{1}\right)$ As we know that the subgraph induced by $A_{1}$ is complete, that is, $K_{2n}$, so all the Type-1, Type-2, and Type-3 edges are exactly the edges of $K_{2n}$, and all the matchings between these edges are counted in Table 6, where $d_{i}^{1}$ denotes the number of matchings of order *i*, where 1≤i≤n;
$\left(d^{2}\right)$ For i=1,2, let $d_{i}^{2}$ indicate the number of order *i* matchings:
  **For**  $\left(d_{1}^{2}\right)$: The number of Type-4 edges that are 4n, which is equal to the number of order one matchings. Consequently, $(d_{1}^{2})=4n;$
  **For**  $\left(d_{2}^{2}\right)$: Suppose $u_{1}∼u_{2}=e$ is a Type-4 edge with $u_{2}\in \Omega$ and $u_{1}\in A_{2}^{j}$ for a fixed 0≤j≤n−1. Then, in addition to the edge *e*, every edge of Type 4 with one end in $A_{2}\backslash \left\{u_{1}\right\}$ and another in $\Omega \backslash \{u_{2}\}$ forms a matching of order two. As a result,
    $$\left(d_{2}^{2}\right)=4\left(n-\frac{1}{2}\right)\left(n\right)=4n\left(n-\frac{1}{2}\right).$$
    Hence, there is no order larger than two matching in this situation;
$\left(d^{3}\right)$ Type 5 has *n* edges, none of which have a similar node. Thus, for any order *i*, there exists a matching such that 1≤i≤n. Suppose $\left(d_{i}^{3}\right)$ represents the number of order *i* matchings. Then, $\left(d_{i}^{3}\right)=\left(\frac{n}{i}\right);$
$\left(d^{4}\right)$ Assume that $\left(d_{i}^{4}\right)$ represents the number of order *i* matchings, where 1≤i≤n+1. Thus, in this context, $(d_{1}^{4})=0$. There are no Type-1 edges that connect a node to any Type-4 edge in $P(Q_{4n})$. Hence, we may obtain a matching in this situation by joining every matching of Type-1 edges to any matching of the Type-4 edges. The edges of Type 1 are also the edges of $K_{2n-2}$, and there are $\left(d_{\ell }^{1}\right)$ matchings of order *ℓ* between them, where 1≤ℓ≤n−1. Every $\left(d_{\ell }^{1}\right)$ can be found in Table 6. In between the edges of Type 4, there are $(d_{1}^{2})=4n$ and $\left(d_{2}^{2}\right)=4n\left(n-\frac{1}{2}\right)$ matchings having one and two orders, respectively.
  As a result of the product rule, we obtain:
  $$d_{2}^{4}=d_{1}^{2}\times d_{1}^{1}=4nd_{1}^{1}.$$
  When 3≤i≤n, then:
  $$\begin{array}{cc} d_{i}^{4} & =d_{1}^{2}\times d_{i-1}^{1}+d_{2}^{2}\times d_{i-2}^{1}\\ & =4nd_{i-1}^{1}+4n\left(n-\frac{1}{2}\right)d_{i-2}^{1}\\ & =2n\left(2d_{i-1}^{1}+2\left(n-\frac{1}{2}\right)d_{i-2}^{1}\right). \end{array}$$
  Furthermore, when i=n+1, then:
  $$d_{i}^{4}=d_{2}^{2}\times d_{i-2}^{1}=4n\left(n-\frac{1}{2}\right)d_{n-1}^{1};$$
$\left(d^{5}\right)$ For i=1,2, $\left(d_{i}^{5}\right)$ represents the total matchings of order *i*. Then, $(d_{1}^{5})=0$. We can only utilize matchings of order one between the edges of Type 4 in this case. Otherwise, we are unable to employ any Type-3 edge, since both kinds of edges often share the nodes in Ω. As a result, we can only obtain matchings of order two in this case. Assume that $N=\{e=u_{1}∼u_{2}\}$ is the order one matching amongst the Type-4 edges with $u_{1}\in A_{2}^{j}$, for 0≤j≤n−1. Then, any Type-3 edge that is non-adjacent with $u_{2}$ can lead to construct an order two matchings. Given that there are 2(n−1) such Type-3 edges, every of which may be utilized in every one of 4n matchings of order one amongst Type-4 edges, so we obtain:
  $$\left(d_{2}^{5}\right)=8n\left(n-1\right);$$
$\left(d^{6}\right)$ For 1≤i≤n, $\left(d_{i}^{6}\right)$, denote the number of order *i* matchings, then $(d_{1}^{6})=0$. To find matchings, both matchings of order one and two between the edges of Type 4 will be considered and any matching of order *ℓ* between the edges of Type 5, where 1≤ℓ≤n−1. Thus, by counting these matchings using the product rule, we obtain:
  $$d_{2}^{6}=1\times 4\times n\times \left(\frac{n-1}{1}\right)=4n\left(n-1\right),$$
  and for 3≤i≤n:
  $$d_{i}^{6}=2n\left\{2\left(\frac{n-1}{i-1}\right)+\left(\frac{n-1}{i-2}\right)+4\left(n-1\right)\left(\frac{n-2}{i-2}\right)\right\};$$
$\left(d^{7}\right)$ Given that the edges of Type 1, Type 2, as well as Type 3 are also the edges of $K_{2n}$, which is induced by $A_{1}$, so we may utilize them to identify matchings between the edges of Type 5 and the edges of $K_{2n}$. Let $d_{i}^{7}$ be the number of order *i* matchings. Then, $d_{i}^{7}=0$. Because no edge of Type 5 shares a node with an edge of $K_{2n}$, this corresponds to each matching of the edges of Type 5. Therefore, each matching of the edges of $K_{2n}$ can be used to determine a match in this situation. Since there exist $d_{\kappa }^{1}$ matchings of the cardinality 1≤κ≤n among the edges of $K_{2n}$, as shown in Table 6, as well as $d_{j}^{3}=\left(\frac{n}{j}\right)$ matchings of the order 1≤j≤n among the Type 5 edges, thus, in this example, the greatest order of a matching is 2n. As a result, we may determine $d_{i}^{7}$, for 2≤i≤2n, as follows:
  $$\begin{array}{cc} d_{2}^{7} & =d_{1}^{1}d_{1}^{3},\\ d_{3}^{7} & =d_{1}^{1}d_{2}^{3}+d_{2}^{1}d_{1}^{3},\\ d_{4}^{7} & =d_{1}^{1}d_{3}^{3}+d_{2}^{1}d_{2}^{3}+d_{3}^{1}d_{1}^{3},\\ & \;\;\vdots \\ d_{i}^{7} & =\sum_{j=1}^{i-1}d_{j}^{1}d_{i-j}^{3}. \end{array}$$
  As a consequence, by the sum rule, the Hosoya index of $P(Q_{4n})$ is as follows:
  $$\begin{array}{cc} 1+\sum_{i=1}^{7}\left(d^{i}\right) & =1+\sum_{i=1}^{n}d_{i}^{1}+\sum_{i=1}^{2}d_{i}^{2}+\sum_{i=1}^{n}d_{i}^{3}+\sum_{i=2}^{n+1}d_{i}^{4}+d_{2}^{5}+\sum_{i=2}^{n}d_{i}^{6}+\sum_{i=2}^{2n}d_{i}^{7}, \end{array}$$
  where:
  $$\begin{array}{cc} d_{i}^{1} & =\frac{1}{i}\prod_{k=0}^{i-1}\left(\frac{2(n-k)}{2}\right),\;d_{1}^{2}=4n,\;d_{2}^{2}=4n\left(n-\frac{1}{2}\right),\;d_{i}^{3}=\left(\frac{n}{i}\right),\\ d_{2}^{4} & =4n\left(\frac{2(n-1)}{2}\right),\\ d_{i}^{4} & =2n\left\{\frac{2}{i-1}\prod_{k=0}^{i-2}\left(\frac{2(n-k-1)}{2}\right)+\frac{2n-1}{i-2}\prod_{k=0}^{i-3}\left(\frac{2(n-k-1)}{2}\right)\right\}, \end{array}$$
  for 3≤i≤n,
  $$\begin{array}{cc} d_{n+1}^{4} & =\frac{2n(2n-1)}{i-2}\prod_{k=0}^{i-3}\left(\frac{2(n-k-1)}{2}\right),\\ d_{2}^{5} & =8n\left(n-\frac{1}{2}\right),\;d_{2}^{6}=4n\left(n-1\right),\\ d_{i}^{6} & =2n\left\{2\left(\frac{n-1}{i-1}\right)+\left(\frac{n-1)}{i-2}\right)+4\left(n-1\right)\left(\frac{n-2}{i-2}\right)\right\},\;\mathrm{for}\;3\leq i\leq n,\\ d_{i}^{7} & =\sum_{j=1}^{i-1}\frac{1}{j}\prod_{k=0}^{j-1}\left(\frac{2(n-k)}{2}\right)\left(\frac{n}{i-j}\right),\;\mathrm{for}\;2\leq i\leq 2n. \end{array}$$

□

**Table 6:** The total non-void matchings in $K_{2n}$.

$K_{2n}$	$d_{1}^{1}$	$d_{2}^{1}$	$d_{3}^{1}$	$d_{4}^{1}$	⋯	$d_{i}^{1}$
$K_{2}$	$\left(\frac{2}{2}\right)$					
$K_{4}$	$\left(\frac{4}{2}\right)$	$\frac{1}{2}\left(\frac{4}{2}\right)\left(\frac{2}{2}\right)$				
$K_{6}$	$\left(\frac{6}{2}\right)$	$\frac{1}{2}\left(\frac{6}{2}\right)\left(\frac{4}{2}\right)$	$\frac{1}{3}\left(\frac{6}{2}\right)\left(\frac{4}{2}\right)\left(\frac{2}{2}\right)$			
$K_{8}$	$\left(\frac{8}{2}\right)$	$\frac{1}{2}\left(\frac{8}{2}\right)\left(\frac{6}{2}\right)$	$\frac{1}{3}\left(\frac{8}{2}\right)\left(\frac{6}{2}\right)\left(\frac{4}{2}\right)$	$\frac{1}{4}\left(\frac{8}{2}\right)\left(\frac{6}{2}\right)\left(\frac{4}{2}\right)\left(\frac{2}{2}\right)$		
⋮	⋮	⋮	⋮	⋮	⋱	⋮
$K_{2n-1}$	$\left(\frac{2n}{2}\right)$	$\frac{1}{2}\left(\frac{2n}{2}\right)\left(\frac{2n-2}{2}\right)$	$\frac{1}{3}\left(\frac{2n}{2}\right)\left(\frac{2n-2}{2}\right)\left(\frac{2n-4}{2}\right)$	$\frac{1}{4}\left(\frac{2n}{2}\right)\left(\frac{2n-2}{2}\right)\left(\frac{2n-4}{2}\right)\left(\frac{2n-6}{2}\right)$	⋯	$\frac{1}{i}\prod_{k=0}^{i-1}\left(\frac{2(n-k)}{2}\right)$

## 6. Concluding Remarks

This work aimed to discuss the structural properties of the power graphs of finite non-abelian groups. In this work, we found the Hosoya properties, that is the Hosoya polynomials, the reciprocal status Hosoya polynomials, and the *Z*-index of the power graphs of certain finite groups. The reciprocal status Hosoya polynomials described in Theorems 1 and 2 are the most notable results of this context. We further illustrated the *Z*-index in Theorems 3 and 4 of the power graphs of the dihedral and the generalized quaternion groups, respectively.

## Data Availability

The data used to support the findings of this study are available within the article.
